# From rings to regions: modeling and mapping climate-driven timber production zones for *Cyclocarya paliurus* by integrating niche models and growth traits

**DOI:** 10.3389/fpls.2025.1635397

**Published:** 2025-07-31

**Authors:** Zijie Zhang, Zhengyang Ye, Xulan Shang, Wanxia Yang, Tongli Wang, Shengzuo Fang

**Affiliations:** ^1^ State Key Laboratory of Tree Genetics and Breeding, Nanjing Forestry University, Nanjing, China; ^2^ Department of Forest and Conservation Sciences, University of British Columbia, Vancouver, BC, Canada; ^3^ National Key Laboratory for the Development and Utilization of Forest Food Resources, Nanjing Forestry University, Nanjing, China; ^4^ Co-Innovation Center for Sustainable Forestry in Southern China, Nanjing Forestry University, Nanjing, China

**Keywords:** wheel wingnut, climate change, Trait-based model, radial growth, wood density, climate-lagged response

## Abstract

**Introduction:**

*Cyclocarya paliurus*, a native hardwood species with multi-functional value, has been prioritized in China’s National Reserve Forest Program. However, uncertainties related to its habitat stability and timber productivity under climate change pose challenges to effective conservation and afforestation planning aligned with national carbon neutrality goals.

**Methods:**

In this study, we constructed species distribution models using Random Forest (RF) and Maximum Entropy (MaxEnt), based on verified field occurrence records and climatic data. Habitat suitability was projected under current and future climate scenarios (SSP2-4.5 and SSP5-8.5). We further analyzed the relationships between climate suitability and growth traits across 27 natural populations.

**Results:**

Both models demonstrated high predictive performance (RF AUC = 0.970, MaxEnt AUC = 0.942), identifying temperature variability and water availability as key limiting factors. Climate suitability was significantly correlated with 20-year diameter growth (R² = 0.625) and wood basic density (R² = 0.463). A stronger correlation was observed between annual growth and climate suitability of the preceding year (R² = 0.695), suggesting a lag effect.

**Discussion:**

By integrating trait–climate relationships, we projected spatial shifts in fast-growing, high-quality timber zones. Future projections suggest a 49.2–60.0% decline in highly suitable habitats and timber forests by the 2050s, with marginal zones shifting northward and toward higher latitudes. This trait-integrated modeling framework offers a scientific basis for climate-resilient conservation and afforestation planning.

## 1 Introduction

Considering the profound impact of global economic expansion in exacerbating climate change, the international community has increasingly acknowledged forest conservation and carbon sequestration as pivotal strategies for climate change mitigation (Zhang et al., 2024a; Anselmetto et al., 2025). In response, China, as a major global emitter, has reiterated its dedication to advancing an environmentally sustainable economic transition, with one key initiative being the continuous emphasis on the National Reserve Forest Project (NRFP), aimed at addressing the national timber supply-demand gap through timber forest cultivation and related measures (Song et al., 2025). Wheel wingnut (*Cyclocarya paliurus*) is an ancient and versatile tree species of the Juglandaceae family, serving as a valuable resource for timber, traditional medicine, and ornamental horticulture in China (Fang et al., 2006). The wood of this species is noted for its high strength, ease of cutting, and smooth surface, with hardness levels surpassing those of most species in the same family (Deng et al., 2014). Due to these exceptional qualities, it has long been a favored material for furniture making, leading to its inclusion in the National Reserve Forest (NRF) (Fang, 2022). So far, the majority of researches on *C. paliurus* has primarily focused on the medicinal properties of its leaves, with little attention given to the cultivation of timber forests.

Driven by practical applications, timber forest cultivation prioritizes growth rate and wood quality, with radial growth and wood density commonly used as key evaluation indicators (Doan et al., 2023; Takahashi et al., 2025). Beyond their silvicultural significance, these traits are also widely employed in climate studies to reconstruct historical climate trends and assess species’ responses to environmental change (Wang et al., 2022, 2025). Accordingly, both genetic background and environmental factors jointly influence these traits, reflecting genotype-environment interactions (Guzmán-Marín et al., 2024; Zheng et al., 2024). To date, several studies have explored the genetic basis of variation in these indicators in *C. paliurus*. For instance, Deng et al. (2014) assessed inter-provenance differences through a field trial and identified superior genetic sources for timber plantation development. Moreover, Fang et al. (2020) reported clear geographic variations in radial growth and wood density, exhibiting distinct latitudinal and longitudinal trends. However, despite these advances in genetic evaluation, the potential impacts of climate change on growth and wood quality in *C. paliurus* remain largely unexplored.

Though now restricted to China, *C. paliurus* once thrived across North America and Europe, as evidenced by fossils dating back approximately 65 million years. The dramatic contraction of its range is largely attributed to global climate shifts over geological time (Burge and Manchester, 2008). Today, with the intensification of climate change, the distribution patterns of more than 17,000 tree species are undergoing notable shifts (Boonman et al., 2024). To cope with these changes, plants primarily rely on migration and adaptation mechanisms (Gray and Brady, 2016; Guo et al., 2019a). However, the dioecious reproductive nature and the fixed reproductive cycle of *C. paliurus* hinder its ability to migrate in the short term (Zhang et al., 2023b). Furthermore, the species demonstrates a high sensitivity to the abiotic stresses caused by climate change, such as drought (Li et al., 2023), soil salinization (Zhang et al., 2024b), and temperature variations (Zhang et al., 2025). Consequently, the species is clearly unable to easily adapt to shifts in its ecological niche. Therefore, after the selection of its superior genotypes, identifying its suitable distribution regions and timber production areas under climate change is of paramount importance for the advancement of *C. paliurus* plantations and serves as a viable solution to achieve the strategic goals of the NRFP.

Species distribution models (SDMs) have become indispensable in biodiversity conservation and ecological research, offering insights into species-environment interactions (Wang et al., 2016; Chen et al., 2024). By integrating species occurrence data with environmental variables, SDMs can predict potential habitat distributions (Zhao et al., 2023; Yang et al., 2024). Among the most prevalent SDM approaches, Random Forest (RF) and Maximum Entropy (MaxEnt) stand out for their robustness and predictive accuracy (Ganglo, 2023; Hu et al., 2025). MaxEnt, relying on the maximum entropy principle, and RF, an ensemble decision tree method, both excel in species distribution prediction with minimal data and assumptions, effectively modeling complex relationships while offering high accuracy and interpretability (Guo et al., 2019b; Feng et al., 2022). Although the predictive results of these two models often differ, most studies tend to use only one of them, and comparative research between the two approaches is less available. In our research, we used these two models to assess their comparative strengths in predicting suitable climatic zones for *C. paliurus*. Their distinct methodological foundations, presence-absence versus presence-only, ensemble-based versus probabilistic-based, enabled us to test the consistency of ecological predictions under different statistical assumptions and to evaluate their ability to reflect spatial variation in growth and wood density. Notably, to the best of our knowledge, no prior study has attempted to model future habitat suitability and timber production zones for *C. paliurus* based on trait-climate relationships. This knowledge gap underscored the need for reliable and high-performing algorithms capable of supporting a trait-integrated modeling framework.

The influence of climate on plant growth often exhibits a lagged effect (Tang et al., 2021; Aguirre-Gutiérrez et al., 2025). Therefore, understanding whether *C. paliurus* shows such delayed responses is critical for optimizing its timber plantation development. In this context, the objectives of this study are to: (1) evaluate the predictive performance of RF and MaxEnt models based on natural distribution records and scale-free climate data; (2) explore the associations between modeled climatic suitability and key growth-related traits across 27 natural populations and determine whether these traits respond to climate with a temporal lag; and (3) integrate the more reliable model with trait-climate relationships to spatially identify and prioritize regions that are most suitable for establishing fast-growing, high-quality timber forests under projected climate change scenarios.

## 2 Materials and methods

### 2.1 Occurrence data

**Figure 1:**
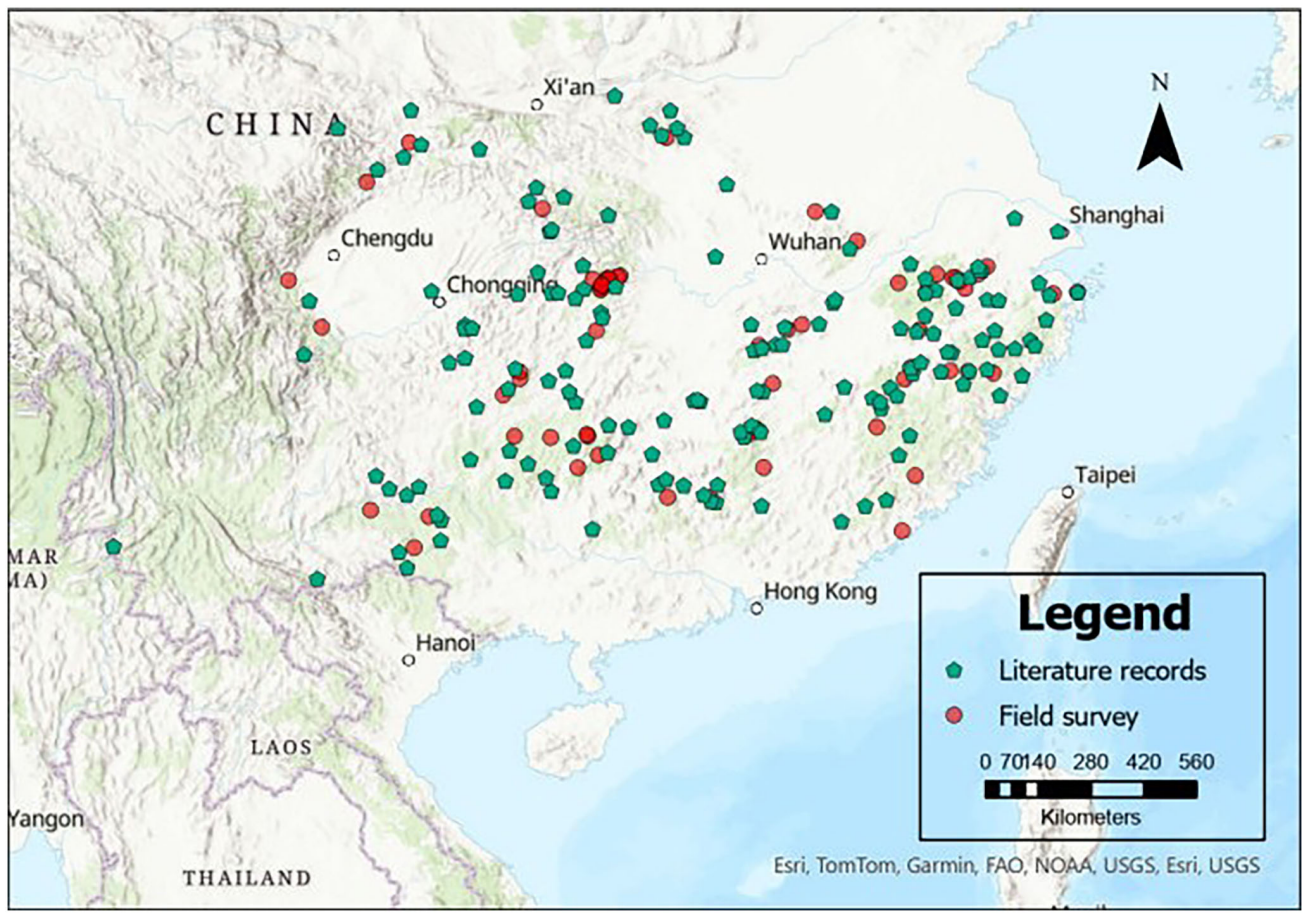
Geographical distribution of *Cyclocarya paliurus* occurrence records. Points with different colors and shapes represent data obtained from different sources.

For model building, a total of 213 naturally occurring *C. paliurus* presence points were compiled from two sources: 150 points were obtained from published literature and three online databases (see **Table 1**), while the remaining 63 points were collected through a field resource survey conducted by our team (**Figure 1**). A spatial thinning process was conducted using a 5-km filtering threshold implemented through the “spThin” package in R (Aiello-Lammens et al., 2015), aiming to control spatial redundancy and sampling bias. The resulting dataset was further validated by manually adjusting coordinates with Google Earth (Hu et al., 2025). These points cover the natural distribution range of *C. paliurus*, which extends from 23°30′ to 33°30′N latitude and 103°30′ to 122°00′E longitude (**Figure 1**). Moreover, the inclusion of pseudo-absence points is crucial for model construction (Wang et al., 2012). To this end, we randomly generated 587 absence points within the species’ distribution range using the “randompoints” function from the “dismo” package in R (Hijmans et al., 2024). Thus, a total of 800 presence and absence points were utilized to develop the SDM.

**Table 1:** Data sources for species occurrence records used in this study.

Full Name	Abbreviation	Website
National Specimen Information Infrastructure	NSII	http://www.nsii.org.cn/
Chinese Virtual Herbarium	CHV	http://www.cvh.org.cn/
Global Biodiversity Information Facility	GBIF	http://www.gbif.org

### 2.2 Climate variables

We used the ClimateAP software to obtain 17 annual climatic variables for 800 locations of the reference period 1961-1990 (**Table 2**). ClimateAP utilizes a dynamic local downscaling approach, allowing for the generation of scale-free climate data (Wang et al., 2017). To assess multicollinearity among these variables, we conducted an iterative VIF-based screening using data from 213 occurrence points. This analysis was carried out with the “regclass” package in R, where we repeatedly refitted linear models and removed the variable with the highest variance inflation factor (VIF) until all remaining variables had VIF values below 10 (Feng et al., 2021). The final set of 17 climatic variables included in our model represents the outcome of this filtering process, ensuring minimal multicollinearity while maintaining ecological relevance. For prediction, the spatial resolution of 800m×800m was used for the normal period of 1961-1990, 2011-2040 (representing the 2020s), and 2041-2070 (the 2050s). Future climate projections were obtained following the method described by Xu et al. (2024) and were based on two greenhouse gas emission scenarios: SSP2-4.5 and SSP5-8.5.

**Table 2:** List of climate variables.

Abbreviation	Variables Description
MAT	Mean Annual Temperature
MWMT	Mean Warmest Month Temperature
MCMT	Mean Coldest Month Temperature
TD	Temperature Difference between MWMT and MCMT
MAP	Mean Annual Precipitation
AHM	Annual Heat: Moisture index (MAT+10)/(MAP/1000)
DD<0	Degree-days below 0°C, Chilling Degree-days
DD>5	Degree-days above 5°C, Growing Degree-days
DD<18	Degree-days below 18°C
DD>18	Degree-days above 18°C
NFFD	The number of Frost-free Days
PAS	Precipitation as Snow (mm) between August in previous Year and July in Current Year
EMT	Extreme Minimum Temperature over 30 years
EXT	Extreme Maximum Temperature over 30 years
Eref	Hargreaves Reference Evaporation
CMD	Hargreaves Climatic Moisture Deficit
RH	Relative Humidity

### 2.3 Habitat model development

MaxEnt and RF models were applied to analyze species suitability and environmental drivers. Modeling was conducted in R Studio 4.1.3 using species occurrence data and 17 climate variables (**Table 2**). The RF model utilized the “randomForest” package (Liaw and Wiener, 2002), while MaxEnt was implemented via “dismo” package (Hijmans et al., 2024). To improve model reliability, highly correlated variables were excluded following Feng et al. (2021). In RF modeling, predictive accuracy was optimized by setting the number of decision trees to 500. A 75/25 split was used for training and validating the RF model, whereas MaxEnt performance was assessed using its internally embedded 10-fold cross-validation framework (Zhang et al., 2023a). To assess model performance, AUC and omission rate were used for MaxEnt, and TSS and AUC for Random Forest. Classification thresholds were followed Feng et al. (2021). We retained MaxEnt’s default settings, including a regularization multiplier of 1.0 and standard feature classes. Although parameter tuning using tools such as ENMeval can further optimize performance, we prioritized consistency and transparency across modeling frameworks. More refined tuning will be considered in future work.

### 2.4 Correlation of climate suitability with radial growth and wood density

In 2014, dominant and healthy individuals were cored from 27 C*. paliurus* sites across its natural range (Fang et al., 2020). These sites were selected from the 63 presence points identified during our field survey, with sampling focused on mixed-species stands containing at least 150 trees ha^-1^. At each site, approximately 15% of the *C. paliurus* trees were sampled, resulting in a total of 215 individuals. The recent 20-year DBH (diameter at breast height) growth, annual DBH growth, and wood basic density were determined using the increment borer method, following the procedures described in our previous study (Fang et al., 2020). For each of the 27 geographic populations, the outermost ring near the bark was regarded as the first year, while the cumulative BDH increment over 20 years and the DBH for each year were recorded as the 20-year DBH growth and annual DBH growth, respectively. Similarly, the average wood basic density corresponding to the recent 20-year growth rings was calculated based on the ring segments. Subsequently, regression analysis was performed to assess the relationships between 20-year DBH growth, wood basic density, and the habitat suitability predictions under the current period. To test the lagged effect, we further analyzed the relationships between previous habitat and annual DBH growth from 1994 to 2014. All the above one-way ANOVA and regression analyses were conducted using SPSS Statistics 29 software.

### 2.5 Planting area prediction for fast-growing and high-quality timber production

We analyzed the relationship of optimal model-predicted habitat suitability to annual DBH growth and average wood basic density for the 27 population sites, with annual DBH growth and wood basic density as dependent variables and habitat suitability as the independent variable, respectively. Following the method described by Feng et al. (2023), the relationship was assessed by fitting models and selecting the optimal model using the trendline package in R. After identifying the best equation, we overlaid suitable habitat maps with spatial predictions of habitat, annual DBH growth, and the wood basic density to estimate potential *C. paliurus* timber production under current and future climates. For visualization and interpretation purposes, habitat suitability scores predicted by the model were grouped into four categories - high (0.6-1.0), medium (0.4-0.6), low (0.2-0.4), and unsuitable (0.0-0.2) - based on commonly adopted thresholds in species distribution modeling (Hu et al., 2025). This stratification was applied exclusively for habitat assessment and did not imply timber suitability. To delineate areas with potential for timber production, we adopted biologically informed criteria derived from field investigations across 27 natural populations. Zones were designated as timber-suitable if they met two empirical thresholds: an annual DBH growth rate exceeding 0.6 cm and a mean wood basic density above 0.5 g cm^-3^. These dual criteria jointly account for both growth performance and wood quality, ensuring that identified regions align with practical forestry standards.

## 3 Results

### 3.1 Model performance evaluation

Both the RF and MaxEnt models demonstrated strong predictive capabilities in modeling species distribution. The RF model achieved a predictive accuracy of 89.97%, with an out-of-bag (OOB) error rate of 10.03% and an AUC of 0.970. Variable importance analysis revealed three primary climatic drivers: TD, MAP, and CMD, with Mean Decrease Accuracy values of 26.33, 24.25, and 22.72, respectively.

The MaxEnt model exhibited excellent discriminatory ability with an AUC value of 0.942 for the training data. The model’s predictive power was primarily driven by four variables: DD<0 contributing 54.3%, CMD at 18.6%, MAP at 11.8%, and TD at 4.9%. These four variables collectively explained 89.6% of the model’s predictive capacity.

Notably, both modeling approaches identified similar key climatic predictors (CMD, MAP, and TD), although their relative importance differed between models. This consistency across different modeling frameworks strengthens confidence in the identified environmental drivers of species distribution. The high performance of metrics for both models (RF accuracy: 89.97%, MaxEnt AUC: 0.942) suggest reliable predictive capabilities for species distribution mapping.

### 3.2 Current habitat distribution of *C. paliurus*

**Figure 2:**
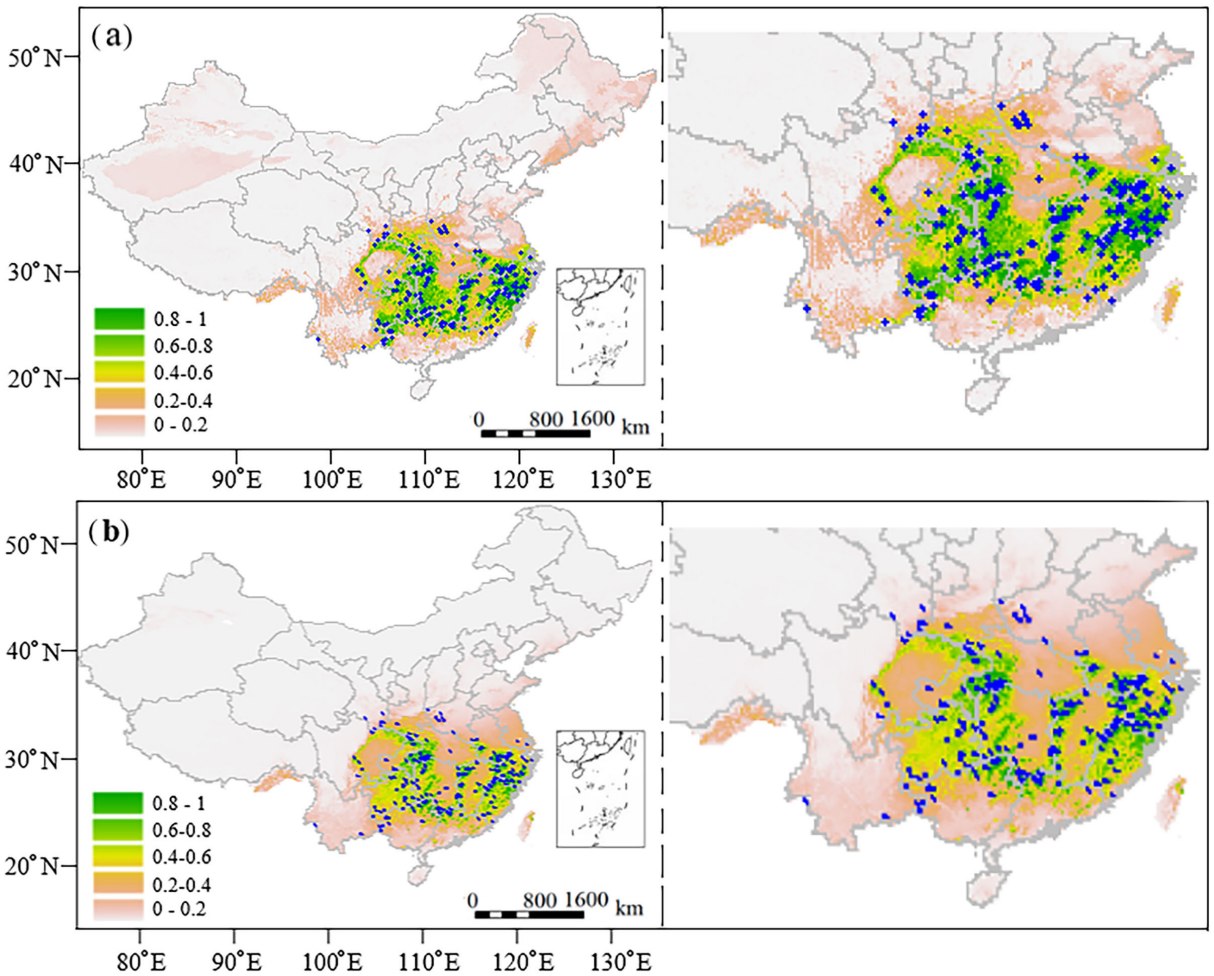
Distributions of the current suitable habitats of *C paliurus* forests in China predicted by **(a)** Random Forest model and **(b)** MaxEnt model. Blue dots indicate the distribution of natural forests.

Both Random Forest (RF) and MaxEnt models were employed to predict the current habitat distribution of *C. paliurus* across China, revealing similar spatial patterns with some variations in area estimates. The RF model projected varying degrees of habitat suitability across China (**Figure 2a**), with highly suitable regions of 740,995 km^2^ (accounting for 7.72% of the total area), moderately suitable regions of 371,154 km^2^, poorly suitable regions of 479,408 km^2^, and unsuitable regions of 8,008,441 km^2^. The highly suitable habitats were predominantly concentrated in provinces characterized by abundant precipitation and humid climates, including eastern provinces Zhejiang, Fujian, and Anhui, central provinces Hunan, Jiangxi, Hubei, and southwestern provinces Guizhou, Guangxi (**Figure 2a**). These regions were primarily distributed along the southern and southeastern coastal areas of China. Moderately and poorly suitable areas typically formed buffer zones around the highly suitable regions (**Figure 2a**).

However, the MaxEnt model showed different areal proportions (**Figure 2b**), which predicted the highly suitable regions, moderately suitable regions, poorly suitable regions and unsuitable regions accounted for 3.90% (374,284 km^2^), 5.02% (482,006 km^2^), 7.44% (714,361 km^2^), and 83.64% (8,029,347 km^2^), respectively. In general, the RF model predicted a larger total suitable area, exceeding the MaxEnt projection by 20,900 km^2^. The most notable difference was in a highly suitable area, where the areas predicted by RF (741,100 km^2^) were nearly double of the MaxEnt prediction (374,400 km^2^). Despite these differences, both models consistently identified the humid southeastern regions as optimal for *C. paliurus* distribution (**Figure 2**).

### 3.3 Geographical variations in growth and wood density

**Figure 3:**
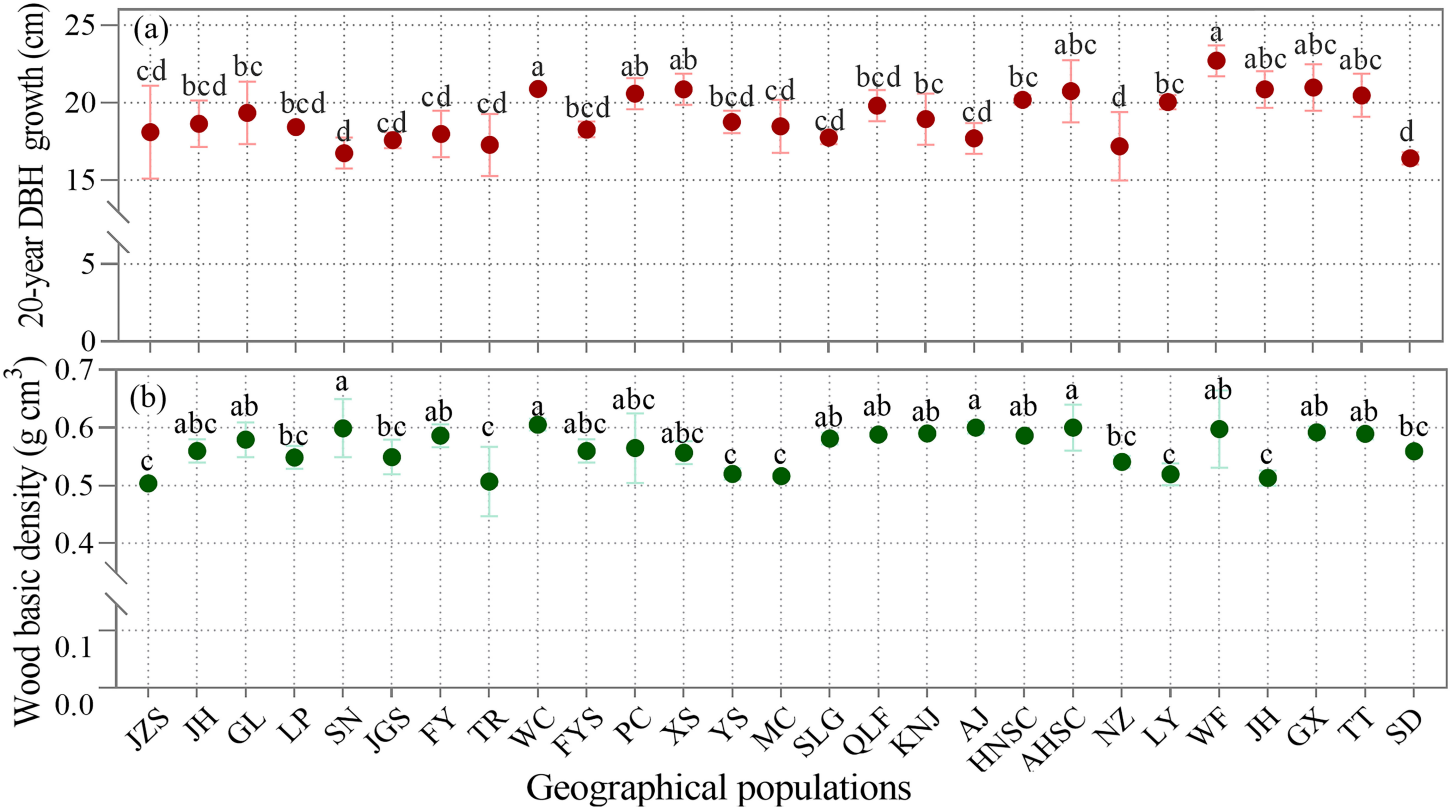
Comparison of **(a)** 20-year diameter growth at breast height DBH growth and **(b)** average wood basic density among the 27 geographical populations of *C paliurus.* Geographical population codes represent sampling locations. Lowercase letters indicate significant differences (*p* < 0.05) among the geographical populations as determined by Duncan’s multiple range test.

Analysis of 27 geographical populations of *C. paliurus* over a 20-year period revealed significant variations (*p* < 0.05) in both 20-year DBH growth and wood basic density (**Figure 3**). The study demonstrated substantial inter-population differences in growth rates, with two populations showing notably superior performance: Wencheng (WC), Zhejiang province and Wufeng (WF), Hubei province (**Figure 3a**). Wood basic density also varied significantly among populations, with the highest values recorded in Anji (AJ), Zhejiang province, and Shucheng (AHSC), Anhui province (**Figure 3b**).

These findings suggest that geographical variation plays a crucial role in determining growth performance and wood properties of *C. paliurus*. However, the observed variations in both growth and wood density also indicate the potential for selective breeding programs to optimize these traits for different end-use applications, which is particularly valuable for future plantation establishment and breeding strategies of *C. paliurus*.

### 3.4 Effects of climate suitability on tree growth and wood density

**Figure 4:**
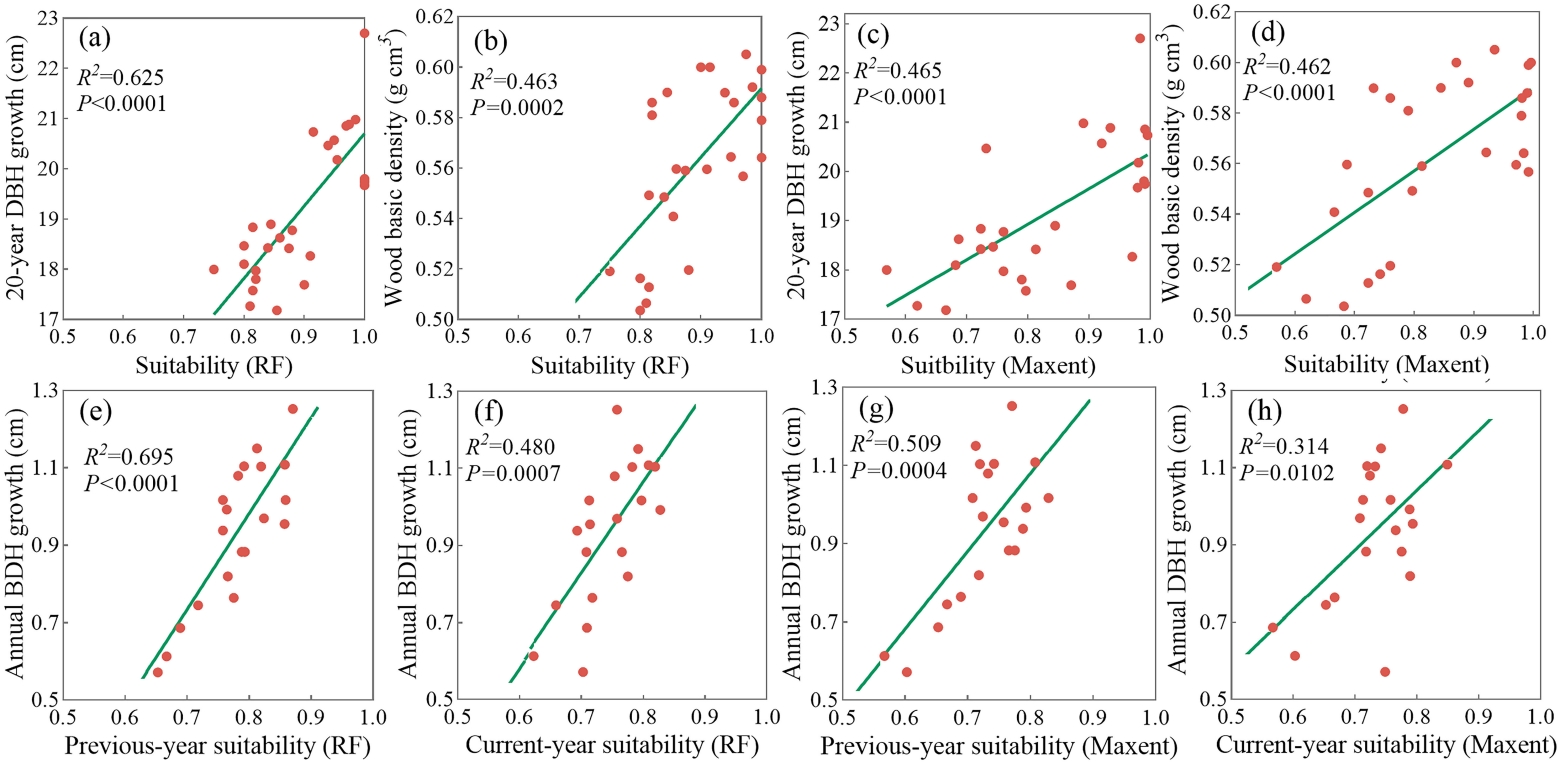
Regression analysis between suitability predicted by two climate models and three tree traits: Current suitability predicted by RF model with **(a)** 20-year DBH growth **(b)** and wood basic density, current suitability predicted by MaxEnt model with **(c)** 20-year DBH growth and **(d)** wood basic density, annual DBH growth with **(e)** annual RF-predicted suitability in the previous year and **(f)** the current year, annual DBH growth with **(g)** annual MaxEnt-predicted suitability in the previous year and **(h)** the current year.

Regression analyses were conducted to assess the relationships of climate suitability to tree growth and wood density in 27 C*. paliurus* populations. Significant positive correlations were found between climate suitability and 20-year DBH growth under both models, with RF showing a stronger association (R^2^ = 0.625, **Figures 4a, c**). Similarly, wood density was positively associated with climate suitability, and the RF model again demonstrated higher explanatory power than MaxEnt (**Figures 4b, d**).

Further analysis revealed a superior correlation between annual DBH growth and the previous-year climate suitability to the current-year suitability, in both RF (R^2^ = 0.695 in **Figure 4e** vs. R^2^ = 0.480 in **Figure 4f**) and MaxEnt (R^2^ = 0.509 in **Figure 4g** vs. R^2^ = 0.314 in **Figure 4h**) models. This finding indicates a lagged climatic effect on radial growth. Overall, RF demonstrated superior alignment with field-investigated traits, particularly in modeling diameter growth and wood density. As a result, RF was selected for following scenario projections and spatial prioritization of optimal timber production zones.

### 3.5 Spatial prediction in suitable habitats and timber production under climate change

**Figure 5:**
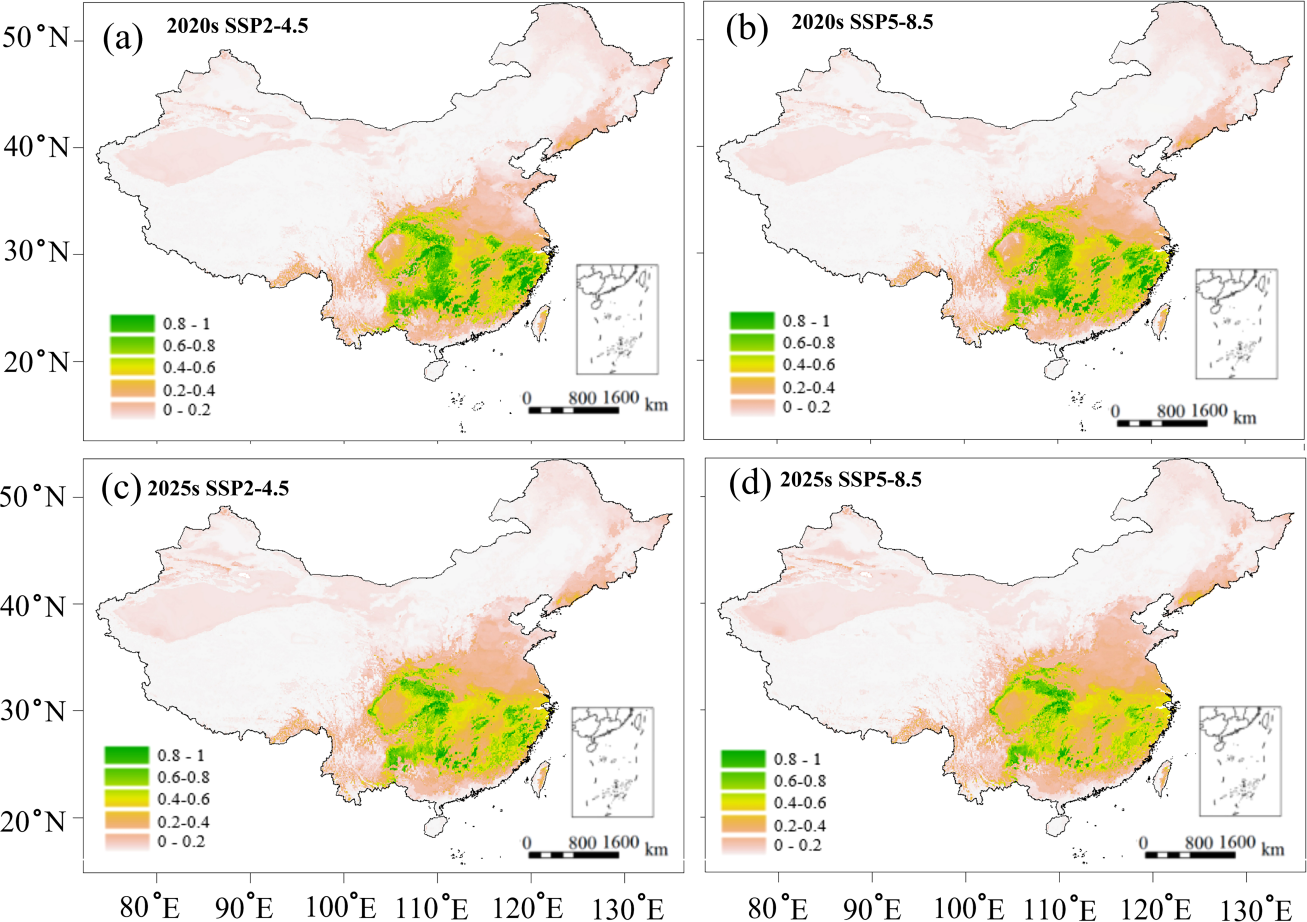
Predicted suitable habitats of *C paliurus* forests based on the SSP2-4.5 and SSP5-8.5 scenarios for two future periods: **(a)** 2020s under SSP2-4.5, **(b)** 2020s under SSP5-8.5, **(c)** 2050s under SSP2-4.5, **(d)** 2050s under SSP5-8.5.

The RF models predicted a significant decrease in the area of highly suitable habitats for *C. paliurus* under future climate scenarios (**Figure 5**). Under the SSP2-4.5 and SSP5-8.5 scenarios, the area of highly suitable habitat by the 2020s was projected to decrease by 31.12% and 32.52% respectively, compared to current conditions (740,994 km^2^) (**Figures 5a, b**). This decline was expected continue, with further reductions of 49.24% and 60.03% projected by the 2050s (**Figures 5c, d**). Conversely, the areas of low and moderate habitat suitability were projected to increase in both the 2020s and 2040s, with a more substantial expansion observed in the low suitability category (**Figure 5**). Under the two scenarios, low suitability habitat was expected to expand by 45.78% and 51.18% in the 2020s (**Figures 5a, b**), respectively, and further increase by 78.13% and 86.48% in the 2050s (**Figures 5c, d**), when compared to current conditions (698,882 km^2^). The area of unsuitable habitat is projected to slightly decrease (1.41% - 3.41%) under all future climate scenarios (**Figure 5**).

**Figure 6:**
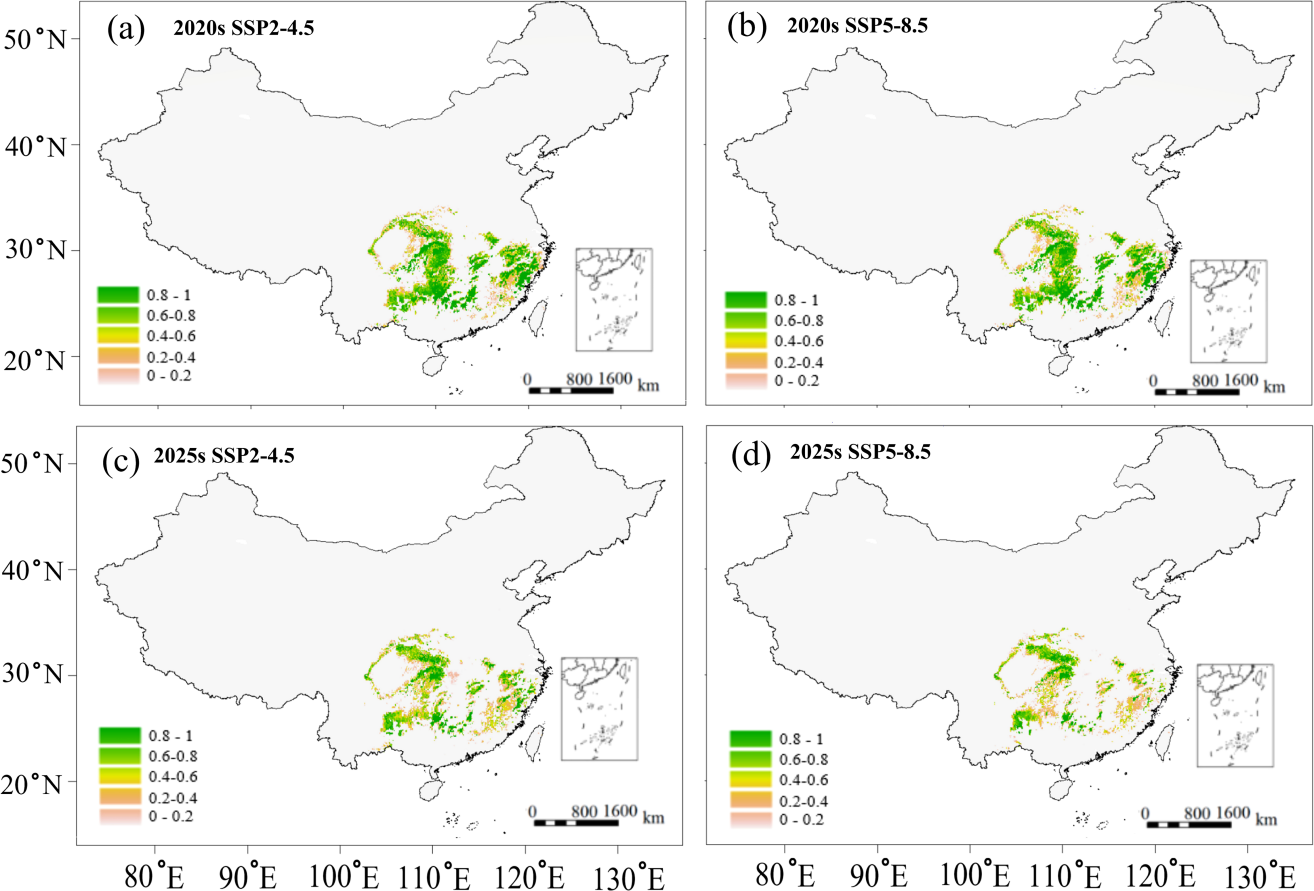
Predicted potential growth region of *C paliurus* based on the SSP2-4.5 and SSP5-8.5 scenarios for two future periods: **(a)** 2020s under SSP2-4.5, **(b)** 2020s under SSP5-8.5, **(c)** 2050s under SSP2-4.5, **(d)** 2050s under SSP5-8.5.

**Figure 7:**
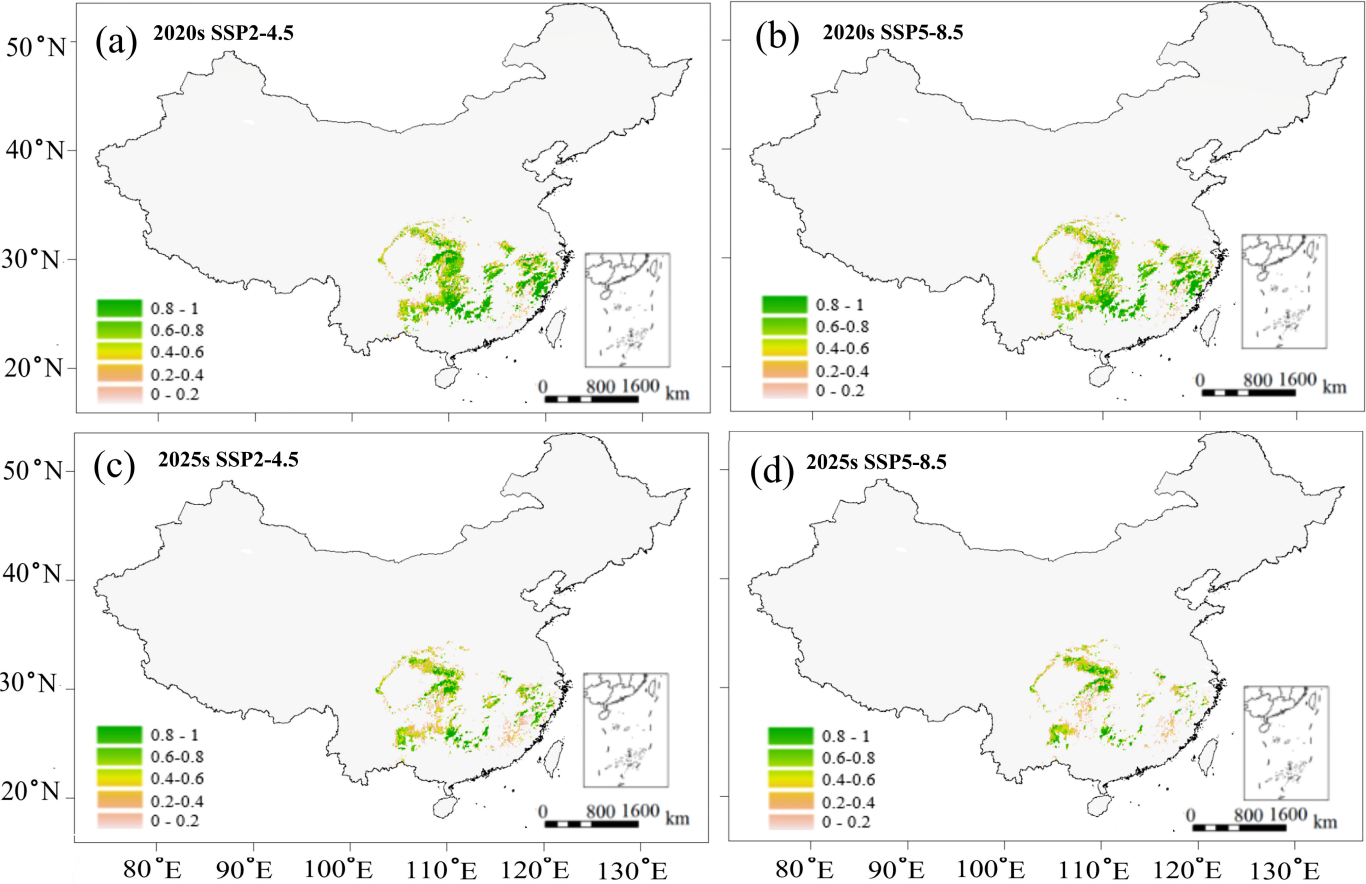
Predicted potential region of *C paliurus* with a high-quality timber based on the SSP2-4.5 and SSP5-8.5 scenarios for two future periods: **(a)** 2020s under SSP2-4.5, **(b)** 2020s under SSP5-8.5, **(c)** 2050s under SSP2-4.5, **(d)** 2050s under SSP5-8.5.

Based on the above correlations (**Figure 4**), we predicted the variations in growth parameter (DBH) and wood quality indicator (wood density) for two future scenarios (**Figures 6**, **7**). Potential growth regions were identified based on the linear relationship between annual climatic suitability and annual DBH growth (**Figure 4e**), whereas the areas that are conducive to producing the high wood quality timber were estimated using the linear correlation between climatic suitability during the reference period (1961-1990) and the wood basic density averaged over a 20-year period (**Figure 4b**). Areas suitable for forest growth (DBH > 0.6) represented a subset of the species’ overall habitat range, indicating that optimal growth conditions are more restrictive than basic survival requirements (**Figure 6**). Furthermore, area capable of supporting wood quality production (wood density > 0.5) were more limited, suggesting that premium wood characteristics require specific environmental conditions (**Figure 7**).

Despite these spatial variations in suitable habitats (**Figure 5**), potential growth regions (**Figure 6**), and high-quality regions (**Figure 7**), the distribution of suitable province remained relatively consistent across all these categories. Climate change projections under both SSP2-4.5 and SSP5-8.5 scenarios indicated a progressive reduction in areas suitable for both growth and high-quality timber regions (**Figures 6**, **7**). This trend suggests that climate change may not only affect the species’ distribution but also impact its commercial timber production potential, highlighting the need for adaptive management strategies in forestry planning.

**Figure 8:**
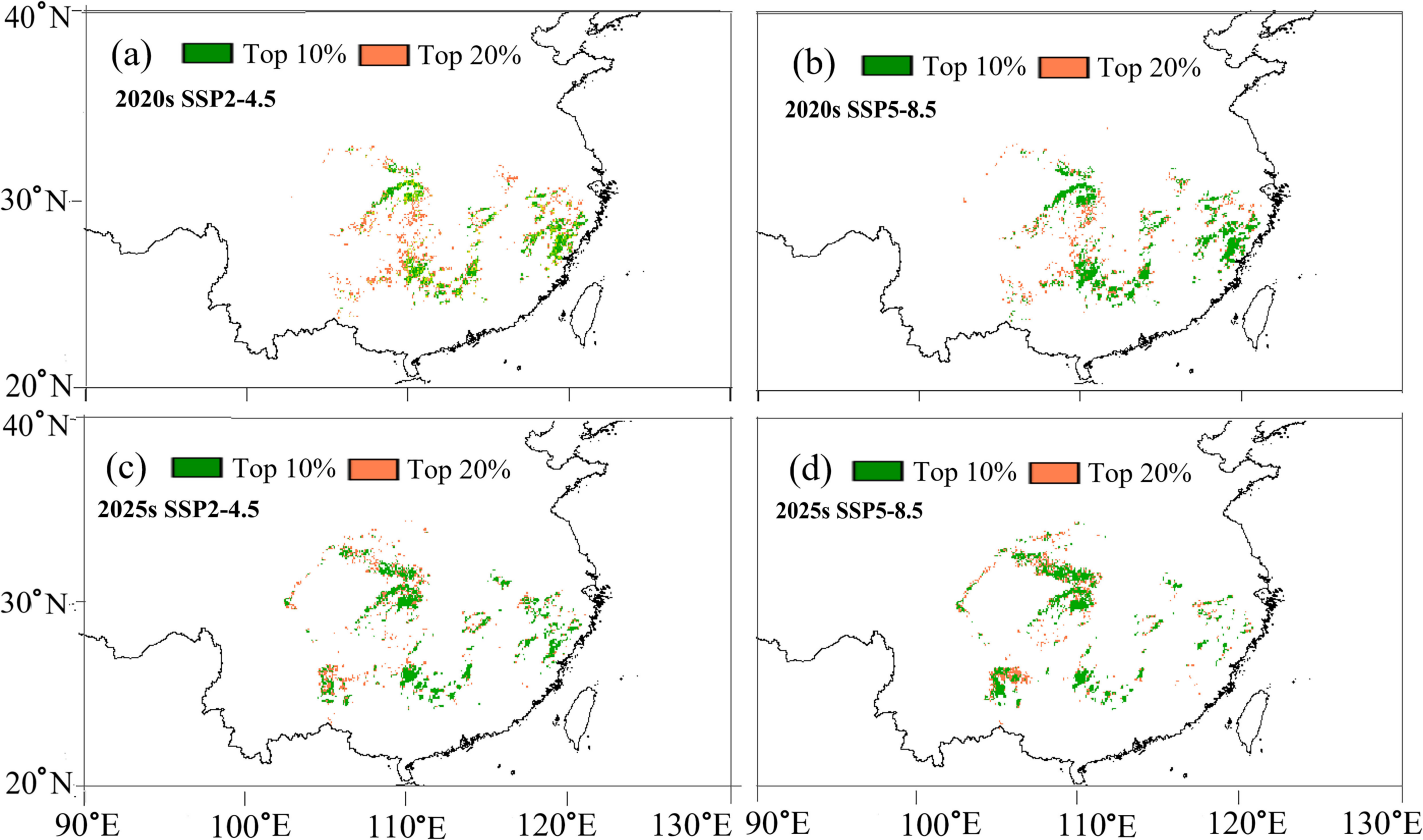
Spatial prediction of optimal *C paliurus* regions for fast-growing and high-quality timber production based on the SSP2-4.5 and SSP5-8.5 scenarios for two future periods: **(a)** 2020s under SSP2-4.5, **(b)** 2020s under SSP5-8.5, **(c)** 2050s under SSP2-4.5, **(d)** 2050s under SSP5-8.5.

Based on these projections, we delineated the optimal (top 10%) and sub-optimal (top 20%) regions for cultivating fast-growing and high-quality timber forests (**Figure 8**). Under both SSP2-4.5 and SSP5-8.5 scenarios, the optimal timber production areas in the 2020s are primarily concentrated in southern Zhejiang, northern Fujian, western Hubei, and the border areas of Hunan, Guangxi, Guangdong, and Jiangxi (**Figure 8**). However, by the 2050s, these areas undergo marked spatial shifts. While parts of western Hubei remain suitable, the optimal zones in Zhejiang and Fujian show significant contraction (**Figure 8**). Notably, new optimal timber production areas emerge in the western part of Guizhou, the northern section of Chongqing, and the central and northern regions of Sichuan (**Figure 8**), likely due to their relatively stable temperature regimes and adequate moisture availability under future climates. Prioritizing emerging regions for afforestation could help maintain timber yield and quality in a changing climate.

## 4 Discussion

### 4.1 Trait-integrated modelling for timber suitability prediction

That machine learning-based SDMs offer tangible advantages over regression-based approaches has been increasingly recognized in ecological modeling, as their capacity to capture complex patterns within occurrence data allows for more precise predictions of potential species distributions (Yu et al., 2020; Bhuyan et al., 2025). The present study demonstrated two widely regarded high-performance algorithms (RF and MaxEnt) show a strong predictive power. However, RF outperformed MaxEnt, as reflected by its higher R^2^ values and stronger correlations with field-observed traits, including DBH growth and wood density. A similar conclusion was also drawn by Zhang et al. (2023b), where RF outperformed MaxEnt in predicting the habitat suitability of *Populus euphratica*, whereas Guo et al. (2018) reported a superior performance of MaxEnt for the same species. This divergence may be partly attributed to differences in sample size, as RF tends to exhibit superior performance with larger datasets (Brown et al., 2023) as used in this study. Meanwhile, RF generally delivers reliable results with its default settings, while MaxEnt often requires time-consuming parameter tuning to achieve optimal accuracy (Zhao et al., 2022). Additionally, the accuracy of the model is significantly enhanced by the high-quality occurrence data, most of which were derived from extensive field surveys, as well as by the inclusion of both presence and absence points, which are equally important for model development (Pearson et al., 2006; Yee and Dirnböck, 2009; Zhang et al., 2023a).

Furthermore, RF better captured the delayed response of radial growth to prior-year climatic conditions, reflecting its capacity to incorporate more complex ecological dynamics (Malla et al., 2023). The enhanced trait-climate congruence observed in RF may stem from its ability to accommodate interactions, nonlinear thresholds, and both presence and absence information (Zhao et al., 2022). In contrast, MaxEnt, despite being widely adopted for presence-only modeling, was comparatively less aligned with variation in growth and structural properties-likely due to its reliance on regularization parameters and simpler response functions (Lissovsky and Dudov, 2021; Chen et al., 2025). Beyond trait relevance, RF also exhibited greater operational efficiency and reduced sensitivity to model tuning. Its built-in variable importance metrics and ensemble learning structure made it more robust in cross-validation scenarios (O’Connell et al., 2025). These advantages collectively justify our decision to adopt RF for downstream habitat projections and timber suitability zoning. Our approach demonstrates how model selection can be grounded in both statistical and biological realism. Comparative model assessment is often overlooked in ecological forecasting studies. By applying and benchmarking both RF and MaxEnt, our study contributes to a more transparent and replicable workflow for integrating distribution modeling with quantitative trait analysis.

Although both models demonstrated strong predictive capabilities, some limitations remain. One key issue is that both models rely solely on climatic variables, while other important factors such as soil conditions, topography, and human disturbance were not included, which may compromise the ecological accuracy of the predictions (Engelhardt et al., 2020). Another concern is the use of pseudo-absence points in RF, which, if randomly assigned without ecological filtering, might introduce bias (Zhao et al., 2023). To enhance model robustness and ecological validity, future efforts should consider incorporating a broader range of abiotic and biotic variables, as well as exploring hybrid or ensemble modeling strategies that combine the strengths of different algorithms.

Functional traits reflect how plants cope with environmental variability, and influence both individual performance and broader patterns of species distribution (Zhou et al., 2014; Díaz et al., 2016). However, conventional SDMs, which are typically grounded in correlations between species occurrence and environmental variables, often overlook these trait-based adaptations (Wang et al., 2016; Zhao et al., 2023). The exclusion of traits such as plant height, wood density or leaf morphology can weaken the connection between modeled habitat suitability and actual ecological performance (Feng et al., 2023; Hu et al., 2025). Although the importance of incorporating functional traits into SDMs is increasingly recognized, some practical limitations still exist. For tree species in particular, available trait datasets remain scarce (Fadrique et al., 2018). The long life cycles and wide geographic ranges of trees make it challenging and costly to obtain observational trait data that span sufficient spatial and temporal scales. As a result, the majority of existing SDMs still rely solely on occurrence data and environmental predictors, with limited integration of trait-based information.

To overcome this limitation, we carried out field surveys across 27 natural populations of *C. paliurus*, covering a wide range of environmental conditions. From these sites, we gathered multi-year data on growth-related traits, such as DBH growth and wood density, which were used to assess the reliability of our SDM. Based on this trait dataset, we designed a two-step modeling strategy aimed at identifying areas suitable for timber production. Firstly, we applied a RF model to generate habitat suitability (**Figure 5**); secondly, these estimates were statistically linked to the observed growth traits data of *C. paliurus* through regression analysis (**Figure 4**). This stepwise method connects climate-based suitability with actual growth performance, enables independent validation and optimization, offers an alternative to costly provenance trials, and increases the reliability of predictions, making it especially valuable for identifying optimal timber production zones for *C. paliurus* plantations.

### 4.2 Key climatic factors affecting *C. paliurus* growth

Both RF and MaxEnt models independently ranked TD, MAP and CMD as primary climate variables in the study, suggesting a consistent climatic signal: temperature variability and moisture availability are fundamental in shaping the distribution of *C. paliurus*. This finding aligns with the ecological behavior of the species as a mesophytic tree native to humid subtropical regions, where warm temperatures and abundant rainfall support its growth (Deng et al., 2015; Yang et al., 2023). TD reflects its sensitivity to thermal fluctuations, especially low winter temperatures can disrupt physiological process, in consistent with the observation that cold conditions significantly suppress *C. paliurus* growth (Zhang et al., 2025). MAP represents the primary water input essential for sustaining transpiration and nutrient transport during the growing season, whereas CMD, as an indicator of drought stress, further constrains plant growth. *C. paliurus* exhibits moderate drought tolerance, however elevated CMD can reduce gas exchange and limit its growth, particularly in marginal habitats (Li et al., 2023). Similar findings have also been reported for other tree species. For instance, Guo et al. (2019b) and Barrio-Anta et al. (2020) respectively modeled the suitable habitat distribution of ginkgo and maritime pine, and reported that both temperature dynamics and water availability are pivotal in regulating their growth and distribution.

Temperature and water availability are key drivers of trait variation in tree species (Rostamikia et al., 2024; Bernal-Escobar et al., 2025; Xue et al., 2025). For instance, a recent study has shown that fluctuations in temperature and water deficit can lead to notable shifts in tropical forest functional traits (Aguirre-Gutiérrez et al., 2025). In our study, both wood density and radial growth of *C. paliurus* exhibited strong linear correlations with climatic suitability, echoing findings from Homeier et al. (2021), where wood and leaf traits were closely linked to MAT. Thus, TD, MAP, and CMD may not only shape species distribution but also likely to influence future patterns of timber production and wood quality in *C. paliurus* plantations.

A particularly intriguing outcome of this study is the observed a lagged response of *C. paliurus* growth to climatic conditions - an aspect that is often underrepresented in SDMs (Zhang et al., 2018). Specifically, we found that the climatic suitability of the preceding year served as a stronger predictor of annual DBH growth than that of the current year. Such a lag effect can be attributed to the carry-over effects of resource availability and physiological processes from the preceding growing season, influencing subsequent year’s growth performance (Babst et al., 2019). For instance, Galiano et al. (2011) reported that carbon reserves accumulated under favorable conditions in one year may sustain growth in the next, even if present-year conditions are less favorable.

### 4.3 Strategic planning for future *C. paliurus* timber production zones

Similar to other tree species such as *Pinus koraiensis* and *Quercus acutissima* (Chen et al., 2024; Guo et al., 2024), SDMs under the SSP5-8.5 scenario predict a marked northward shift in both the suitable distribution range and timber production zones of *C. paliurus*. In particular, future climatic conditions are expected to reduce habitat suitability in southern and southeastern coastal regions, where prolonged heat stress, increased water deficits caused by excessive transpiration, more frequent drought events, and intensified salinity stress may collectively exceed the species’ ecological tolerance thresholds (Sadok et al., 2021; Chen et al., 2025).

To adapt to these changes, a combination of strategic and operational interventions should be considered. At the regional scale, afforestation strategies need to be spatially reoriented, with greater emphasis placed on the anticipated expansion zones (Hubei, Guizhou, Chongqing, Sichuan) in China. At the same time, safeguarding genetic diversity is essential—not only through *in situ* conservation, but also via the systematic collection of germplasm from existing populations to facilitate long-term breeding programs (Salgotra and Chauhan, 2023). Developing genotypes with an enhanced tolerance to drought stress would be key to maintaining productivity under future climate extremes (Brodribb et al., 2020). Third, fostering structurally and functionally diverse forest systems (such as mixed-species plantations), which can provide greater ecological buffering capacity, and improve forest resilience in the face of increasing climatic volatility (Liu et al., 2018). On-the-ground implementation also requires flexible and climate-responsive management. In stable core regions, techniques like selective thinning and microclimate regulation—e.g., preserving understory cover or improving soil moisture retention—can help sustain growth under warming conditions (Kaarakka et al., 2021). In ecotonal or declining zones, adaptive silvicultural practices such as dynamic rotation ages or assisted migration of resilient genotypes may mitigate productivity losses (Gustafson et al., 2020). Additionally, the integration of remote sensing tools with real-time environmental monitoring can support responsive decision-making and enable early-warning systems for risk management (Xi et al., 2024). Together, these multi-scale strategies can improve the adaptive capacity of *C. paliurus* plantations in the face of ongoing climate change.

## 5 Conclusion

In light of escalating climate challenges and national reforestation strategies, this study systematically investigates the spatial and temporal patterns of habitat suitability, wood production potential, and climatic sensitivity of *C. paliurus* by RF and MaxEnt modeling approaches. Both two approaches showed a high predictive performance, while the RF modeling approach outperform the MaxEnt in predicting current habitat suitability. Climatic suitability showed strong correlations with tree growth traits, with lagged climate effects indicating stronger growth responses to preceding year conditions. Under future climate scenarios, a significant contraction in highly suitable areas is projected for *C. paliurus*, especially under high-emission pathways. Strategic zoning further identifies emerging regions (particularly in western and northern provinces in China) as its potential areas for fast-growing and high-quality timber production. Overall, our findings highlight the value of integrating climate modeling with trait-based analysis to guide climate-resilient forestry planning, and would offer a theoretical reference for national reserve forest planning of other tree species in China.

## Data Availability

The original contributions presented in the study are included in the article/supplementary material. Further inquiries can be directed to the corresponding author/s.
